# Supplementary material for: Splice-Junction-Based Mapping of Alternative Isoforms in the Human Proteome
Source: Cell Rep. Author manuscript; Available in PMC 2020 Jan 15. (PMC6961840; doi:10.1016/j.celrep.2019.11.026)
Supplement: 2 [file NIHMS1546469-supplement-2.zip › DF1/PXD010154/AdrenalGland/AdrenalGland_6_RBMS1_MITQTSITPYIASPVSAYQVAK.pdf]

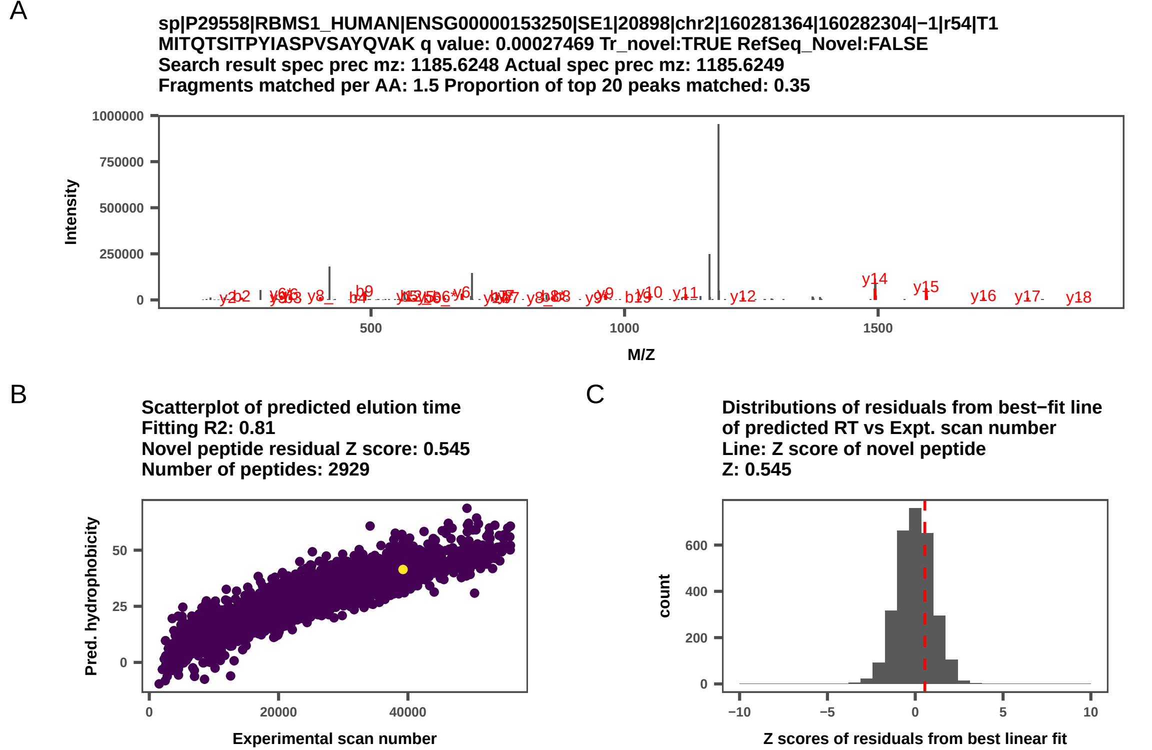
A
sp|P29558|RBMS1_HUMAN|ENSG00000153250|SE1|20898|chr2|160281364|160282304|−1|r54|T1
MITQTSITPYIASPVSAYQVAK q value: 0.00027469 Tr_novel:TRUE RefSeq_Novel:FALSE
Search result spec prec mz: 1185.6248 Actual spec prec mz: 1185.6249
Fragments matched per AA: 1.5 Proportion of top 20 peaks matched: 0.35
Intensity
1000000
750000
500000
250000
0
y12
y14
y15
y16
y17
y18
500
1000
1500
M/Z
B
Scatterplot of predicted elution time
Fitting R2: 0.81
Novel peptide residual Z score: 0.545
Number of peptides: 2929
Pred. hydrophobicity
50
25
0
20000
40000
Experimental scan number
C
Distributions of residuals from best−fit line
of predicted RT vs Expt. scan number
Line: Z score of novel peptide
Z: 0.545
count
600
400
200
0
−10
−5
5
10
Z scores of residuals from best linear fit
